# Infection and migration incidence of cardiac implantable electrical devices in Japan: Web‐based survey results

**DOI:** 10.1002/joa3.12345

**Published:** 2020-04-21

**Authors:** Hiroshi Nakajima, Nobuhiro Nishii

**Affiliations:** ^1^ Chiba‐Nishi General Hospital Matsudo Japan; ^2^ Okayama University Hospital Okayama Japan

**Keywords:** CIEDs, device migration, infection, survey

## Abstract

Web‐based survey was conducted for experiences of cardiac implantable electrical device (CIED) infection and migration in Japan. A total of 155 cardiologists’ answer was collected in January, 2018. CIED includes pacemaker (PM), implantable cardioverter defibrillator (ICD), cardiac resynchronization therapy pacemaker (CRT‐P), and cardiac resynchronization therapy ICD (CRT‐D) and total of 10,499 CIEDs’ experiences of within previous twelve months were reported. CIED includes pacemaker (PM), implantable cardioverter defibrillator (ICD), cardiac resynchronization therapy pacemaker (CRT‐P), and cardiac resynchronization therapy ICD (CRT‐D.). The infection rate of PM, ICD, CRT‐P, and CRT‐D was 0.79%, 0.81%, 0.45%, and 2.0%, respectively, and the device migration rate was 0.68%, 0.64%, 0.45%, and 0.93%, respectively. The overall infection rate was 0.85% and migration rate was 0.68%.

## 1 INTRODUCTION

We reported in 2016 on cardiac implantable electrical device (CIED) infection rate in Japan and it showed an overall infection rate of 1.12%.^1^ The report was based on the interviews of the device implanters across Japan. Adding to that report, we seek cost effective and repetitive approach utilizing the Internet platform to access the CIED implanters across Japan. The infection and device migration are significant adverse effects for the CIED patients but not many evidences are available for Japanese patient population and medical environment. The significance of the event affects patients’ mobility and mortality, length of hospitalization, and healthcare cost.^2^, ^3^, ^4^ Additionally, the status of application of prophylactic strategies in Japan where various antibiotic prophylactic regimens and surgical practice were mentioned in various consensus statements,^5^, ^6^ is not clear. Thus, adding another data on CIED infection and device migration in Japan would be valuable to those who perform CIED implants.

## 2 METHOD

A physician panel of Macromill Carenet Inc which consisted of more than six thousand cardiologists was utilized and the target panel was narrowed to CIED implanters and asked questions on CIED infection and migration. The web‐based questions were asked in a way such as “Please let us know how many cases you experienced device infection in recent one year?” and “For the infected patients what was the treatment? Please select from below with number of patients.” In general, the questions were formulated in order to know the experience of the respondents, but not each case's clinical detail. The definition of the infection was: “The case which patient feels hot sensation, redness, and pain, and needed administration of antibacterial agent or prolongation of it, and/or needed surgical intervention such as re‐implantation.” The definition of migration was: “The cases where device system was affected such as damage or dislodgement of the lead, and/or patient needed medical treatment. The questionnaire was open for two days for the panel. A total of 232 doctors answered to it, and 77 were excluded because they were not implanting CIEDs. A total of 155 answers were included in this report. The questions were asked such a way as “in your institute…”, and it was confirmed that all the respondents were from different institute therefore representing unique individual hospital in this report.

## 3 RESULTS

Results are shown in Tables 1, 2, 3, 4, 5, 6, 7, 8. Tables 1 and 2 summarized the characteristics of institution which respondents were from. Most of the respondents belonged in Cardiology department and half of them performed all the CIED implant and the half performed PM implants only. Majority (65%) of institutes were medium‐ to large‐sized hospitals according to number of beds. The implant was mostly (79%) performed at Cath. Lab, and experience years of implanting physicians was relatively long (more than 10 years: 53%). The geographical coverage was wide (91.3% of all prefectures) and among 46 prefectures, four prefectures, for example, Fukushima, Nara, Shimane, and Okinawa were not covered. The most concentrated area was Tokyo and the ratio was 10.3%. The Implants were mostly (79%) performed at cath. Lab., and experience years of implanting physicians was relatively long (more than 10 years: 53%). Infection and device migration rate are shown in Table 3. Infection was categorized into “definitive” and “in‐definitive” because oftentimes it is difficult to reach to the definitive diagnosis.^7^ In this circumstance, the infection rate including in‐definitive ones was reported. The overall infection rate including in‐definitive diagnosis was 2.0%. As a reference, Table 3 includes infection rate from recent report from Olsen et al^8^ from Danish device‐cohort. Table 4 shows preventive measures prior or during implant procedure and administering direct and/or indirect antibiotics were main method followed by pocket cleansing. As the treatment of infection, invasive interventions were taken in 60% and it is significant event for the patients. Among various medical aspects of infection and migration, this is the first report in Japan which showed days of hospitalization and days to device replacement after device infection was diagnosed and is presented in Table 6. This suggested that when infection has occurred, significant hospital stay was needed and therefore suggesting healthcare burden may increase. Tables 7 and 8 show device migration consequences and its treatment. The major consequence of device migration is lead dislodgement (51%) and invasive treatment was needed in 39% of cases.

**TABLE 1:** Institution of respondents (155 sites)

Clinical department		Implanting CIED at institute		Number of beds	
Cardiology 134	(86.5%)	All CIEDs	80 (51%)	20‐99	7 (5%)
Cardiac surgery	1(0.6%)	PM only (<20 annually)	29 (19%)	100‐199	24 (15%)
Cardio vascular surgery	20 (12.9%)	PM only (≧20 annually)	46 (30%)	200‐299	23 (15%)
				300‐399	38 (24%)
				400‐499	18 (12%)
				≧500	45 (29%)

Abbreviation: CIED, cardiac implantable electrical device.

**TABLE 2:** Characteristics of implants (155 sites)

Number of implants		Implant facility		Experience years of implanter (n = 763)	
PM initial	5348	Cath. lab.	122 (79%)	>3	122 (16%)
PM replacement	2779	Operating room	33 (21%)	3‐10	237 (31%)
ICD initial	832			>10	404 (53%)
ICD replacement	402				
CRT‐P initial	310				
CRT‐P replacement	133				
CRT‐D initial	463				
CRT‐D replacement	182				

Abbreviation: CRT‐D, cardiac resynchronization therapy ICD; CRT‐P, cardiac resynchronization therapy pacemaker; ICD, implantable cardioverter defibrillator.

**TABLE 3:** Infection and device migration (155 sites) and reference from Danish Device‐Cohort

CIED type	Number of implants	Infection (definitive)	Infection (incl. suspicious cases)	Device migration
PM	8127	64 (0.79％)	158 (1.9%)	56 (0.68%)
ICD	1234	10 (0.81％)	25 (2.0%)	8 (0.64%)
CRT‐P	443	2 (0.45％)	8 (1.8%)	2 (0.45%)
CRT‐D	645	13 (2.0%)	27 (4.2%)	6 (0.93%)
Total	10 499	89 (0.85%)	218 (2.0%)	72 (0.68%)
Reference: Danish Device‐Cohort (1982‐2018)				
PM	100 374	1194 (1.19%)	—	—
ICD	16 718	320 (1.91%)	—	—
CRT‐P	4630	101 (2.18%)	—	—
CRT‐D	6323	212 (3.35%)	—	—
Total	128 045	1827 (1.43%)	—	—

Abbreviation: CIED, cardiac implantable electrical device; CRT‐P, cardiac resynchronization therapy pacemaker; CRT‐D, cardiac resynchronization therapy ICD; ICD, implantable cardioverter defibrillator.

**TABLE 4:** Prevention of infection (155 sites, multiple responses)

Preventive measures for infection	
Indirect antibiotics administration	122 (79%)
Cleansing the pocket	117 (75%)
Subcutaneous implant	54 (35%)
Direct antibiotics administration in the pocket	46 (65%)
Preoperative checking such as MRSA	38 (25%)
Use of medical adhesive	12 (8%)
Use of antibacterial tape	10 (6%)
Other	3 (2%)

**TABLE 5:** Treatment of infection (Data from available 218 cases)

Treatment	
Administration/prolongation of antibiotics treatment	85 (39%)
Entire system removal (device and leads)	65 (30%)
Device removal	46 (21%)
Reoperation for modification	20 (9%)
Other	2 (1%)

**TABLE 6:** Duration of hospitalization and number of days to device replacement due to infection (Data from available 95 cases)

CIED type	Hospitalization days	Days to device replacement
All	Average: 26.2 days (Min = 3, Max = 100, SD = 16.1)	20.5 days (Min = 1. Max = 80, SD = 14.2)

Abbreviation: CIED, cardiac implantable electrical device.

**TABLE 7:** Complication by device migration (data from available 69 cases)

CIED type	Lead dislodgement	Twiddler's syndrome	No complication
All	35 (51%)	3 (4%)	31 (45%)

Abbreviation: CIED, cardiac implantable electrical device.

**TABLE 8:** Treatment of device migration (data from available 72 cases)

Treatment	
Continuous observation	23 (32%)
Device removal	19 (26%)
Device reprograming	16 (22%)
Entire system removal (device and leads)	9 (13%)
Other	5 (7%)

## 4 CONCLUSION

The current status of infection, device migration, prophylactic measures, and post incident treatments among CIED patients were reported. Adding to that, utility of web‐based survey for real world medical environment in Japan was shown. The data presented in this report were snapshots of current medical practice and may not be suitable for further analysis for scientific conclusion, however, it may serve as one of the basic data of device infection and migration in Japan. Limitation of this report is that since the respondents were voluntarily answered according to their experience, personal bias may be introduced in the results.

## Supporting information

Data S1
